# Early risk factors for joint trajectories of bullying victimisation and perpetration

**DOI:** 10.1007/s00787-022-01989-6

**Published:** 2022-04-25

**Authors:** Athena R. W. Chow, Jean-Baptiste Pingault, Jessie R. Baldwin

**Affiliations:** 1 Department of Clinical, Educational and Health Psychology, Division of Psychology and Language Sciences, University College London, London, UK; 2 Social, Genetic and Developmental Psychiatry Centre, Institute of Psychiatry, Psychology and Neuroscience, King’s College London, London, UK

**Keywords:** Bullying, Victimisation, Bully victims, Joint trajectories, Risk factors, Longitudinal studies

## Abstract

Bullying victimisation is a prevalent stressor associated with serious health problems. To inform intervention strategies, it is important to understand children’s patterns of involvement in bullying victimisation and perpetration across development, and identify early risk factors for these developmental trajectories. We analysed data from the Millennium Cohort Study (*N* = 14,525; 48.6% female, 82.6% White), a representative birth cohort of British children born in 2000–2002 across the UK. Bullying victimisation and perpetration were assessed via child, mother, and teacher reports at ages 5, 7, 11, and 14 years. Early risk factors (child emotional, cognitive, and physical vulnerabilities, and adverse family environments) were assessed at ages 9 months, 3, and 5 years. Using k-means for longitudinal data, we identified five joint trajectories of victimisation and perpetration across ages 5, 7, 11, and 14: uninvolved children (59.78%), early child victims (9.96%), early adolescent victims (15.07%), early child bullies (8.01%), and bully- victims (7.19%). Individual vulnerabilities (e.g., emotional dysregulation, cognitive difficulties) and adverse family environments (maternal psychopathology, low income) in pre-school years independently forecast multiple trajectories of bullying involvement. Compared to victims, bully-victims were more likely to be male, have cognitive difficulties, and experience harsh discipline and low income. Interventions addressing these risk factors (e.g., via accessible mental health care, stigma-based interventions, or programs to support low-income families) may help to prevent bullying involvement and its associated sequelae.

**Supplementary Information:**

The online version contains supplementary material available at 10.1007/s00787-022-01989-6.

## Introduction

Bullying victimisation is a global public health concern [1]. Victims of bullying are at risk for mental health problems, such as anxiety, depression, and self-harm [2–4] and physical health problems, such as obesity and increased inflammation [5]. Bullies are likely to exhibit conduct problems and delinquency [6], while bully-victims (children who bully others and are victimised) have the highest risk of health problems compared to “pure” victims and bullies [7]. However, bullying involvement is dynamic and changes over time for different children [8]. To inform preventative interventions, it is important to understand children’s developmental patterns of victimisation and perpetration, and identify early risk factors for these trajectories.

Longitudinal studies have shown individual differences in the timing and chronicity of bullying victimisation and perpetration from childhood to adolescence. For example, some children are victimised in childhood only, others in adolescence only, and some chronically over time [9–11]. Similarly, children who bully others also have varying trajectories, either increasing, decreasing, or chronically perpetrating bullying from childhood to adolescence [6, 8, 12]. In addition, because many children who are victimised also bully others [13, 14], it is critical to understand the joint development of bullying victimisation and perpetration over time. To date, only a handful of studies based in Scotland [12], Canada [8], Australia [15], South Korea [16, 17], and China [18] have mapped the joint developmental trajectories of bullying victimisation and perpetration. These studies have broadly provided evidence for three to six joint trajectories, which typically include an uninvolved group, an increasing victimisation group, a decreasing victimisation group, a bully group, and a bully-victim group [8, 12, 15–18]. However, these studies examined bullying involvement over a short period of three to four years, focusing on early to mid-adolescence only [8, 12, 16, 18] or mid to late adolescence only [15, 17]. Little is known about the joint trajectories of bullying victimisation and perpetration over a longer period from early childhood across adolescence.

Identifying early risk factors for developmental trajectories of victimisation and perpetration could inform interventions to prevent children becoming involved in bullying. This is important because current anti-bullying interventions do not prevent all victimisation and perpetration [19], suggesting that complementary approaches to support vulnerable children are needed. Evidence suggests that early child and family factors are associated with bullying involvement. For example, male sex [20], low birth weight [21], cognitive difficulties [22], emotional dysregulation [23], obesity [20], and long-term health problems [24] have been associated with bullying victimisation and perpetration. Evidence also suggests that victims and bullies are more likely to come from low-income families [25] and experience negative parenting, such as harsh discipline [14] and parental depression [13]. Notably, research has suggested that bully victims may experience greater familial adversity than victims or bullies [13, 14]. However, the majority of this past research on risk factors for bullying was cross-sectional [26], looked at only bullies and victims but not bully-victims [20], or used a-priori defined categories of bullying involvement [13], which may not reflect actual developmental patterns. Though a few notable studies have examined risk factors for longitudinal trajectories of bullying victimisation [11] or perpetration [6], early life risk factors for *joint* trajectories of victimisation and perpetration have not been identified. Therefore, it is not known whether distinct early life factors forecast following a bully-victim trajectory versus a “pure” victim or bully trajectory.

To address these research gaps, we examined early risk factors for joint trajectories of bullying involvement from early childhood to adolescence in a large UK-based cohort (*N* = 14,525). Specifically, our study aimed to (1) map the joint trajectories of bullying victimisation and perpetration from childhood to adolescence, (2) identify early child and family factors associated with joint trajectories of bullying involvement, and (3) examine whether bully-victims had distinct risk factors to victims and bullies.

## Methods

### Participants

Participants were members from the Millennium Cohort Study (MCS), an ongoing longitudinal birth cohort study following a representative sample of 18,552 children (48.6% females) born between 2000 and 2002 in the UK (England, Scotland, Wales and Northern Ireland). To ensure adequate representation, the MCS implemented disproportionate stratification to over-represent areas with high ethnic minority proportions, residents of high poverty areas, and residents of the three smaller UK countries [27]. Detailed information on the MCS can be found in the technical reports (https://cls.ucl.ac.uk/cls-studies/millennium-cohort-study/). This study analysed data from 14,525 children from sweeps 1 to 6 (ages 9 months, 3, 5, 7, 11, and 14 years) after multiple imputation (minimum N in the complete case sample = 11,338). The ethnic distribution of the sample included 82.6% children of White British, White Irish and any other White background, 6.83% children of Pakistani and Bangladeshi origin, 3.60% Black or Black British children, 2.99% of Mixed ethnicity, 2.51% Indian, and 1.44% reported to be from an “Other Ethnic group (inc. Chinese, Other)”. Access to MCS data is publicly available through the UK Data Service. The NHS Research Ethics Committee provided ethical approval for all sweeps. This study conforms to the STROBE reporting guidelines for cohort studies (Table S1 in the supplementary materials).

### Measures

#### Bullying involvement

Bullying victimisation and perpetration were assessed at ages 5, 7, 11, and 14 using reports from children, mothers, and teachers (see Table S2 for details). These assessments capture experiences of bullying throughout primary school (i.e., age 5 to 11) to the middle of secondary school (i.e., age 14) in the British education system. Where possible, we included information from multiple informants to maximise capture of bullying [28], given that different informants have access to different information (e.g., children have cross-context knowledge of bullying, teachers witness bullying at school, and parents witness it at home), and may be affected by different biases (e.g., self-reports from children may be susceptible to social desirability bias) [29]. Indeed, consistent with previous research [28], we observed low to moderate correlations in reports of bullying between informants (Tables S3–S4), suggesting that each informant contributes a unique perspective on the child’s bullying involvement. Children self-reported on bullying at ages 7, 11, and 14; parents reported on bullying at ages 5, 7, and 14, and teachers reported on bullying at ages 7 and 11. For each time point, we averaged the scores across informants to derive mean composite scores for victimisation and perpetration, as recommended by previous research [28, 30, 31].

At age 7, children were asked “How often do other children bully you?” for victimisation, and “How often are you horrible to other children at school?” for perpetration, for which they could respond with “never”, “some of the time”, or “all of the time”. At ages 11 and 14 years, children were asked “How often do other children hurt you or pick on you on purpose?” for victimisation, and “How often do you hurt or pick on other children on purpose?” for perpetration, which they responded to on a six-point scale (“never”, “less often”, “every few months”, “about once a month”, “about once a week”, “most days”). The Peer Problems Scale from the Strengths and Difficulties Questionnaire (SDQ) [32] was used to measure bullying from mother reports at ages 5, 7, and 14 years, and teacher reports at ages 7 and 11 years. Victimisation was measured by asking whether the child “is picked on or bullied by other children”, while perpetration was measured by asking whether the child “fights with or bullies other children”. Mothers and teachers rated the extent to which the statement applied to the child (“not true”, “somewhat true”, “certainly true”). Further details showing the item coding, descriptive statistics, and correlations between the bullying measures are available in the supplementary materials (Tables S2, S3, S4).

#### Early risk factors

We selected early risk factors on the basis of meta-analytic evidence [20] as well as the available early measures collected by the MCS. Details on the assessment of all early life risk factors are available in Table S5. Child factors included sex, birthweight, and infant temperament (all assessed at 9 months), BMI, physical health problems (e.g., asthma, dermatitis), independence and self-regulation, and emotional dysregulation (assessed at ages 3 and 5), and cognitive ability (assessed at age 5). Family factors included family income (assessed at 9 months), mother–child relationship, family size (both assessed at age 3), maternal discipline style, and maternal mental health (assessed at ages 3 and 5).

### Statistical analyses

Analyses were conducted in R version 3.6.1 (R Core Team, 2019) and the script is available online (https://osf.io/g5928).

*Joint developmental trajectories of bullying victimisation and perpetration.* To estimate the joint trajectories of bullying victimisation and perpetration across childhood and adolescence, we used k-means for longitudinal data with the *KmL3D* package [33]. Each observation (each individual’s average composite victimisation and perpetration scores at ages 5, 7, 11, and 14) was assigned to one of two clusters. Next, each participant was reassigned iteratively to the closest cluster, yielding two joint developmental trajectories of victimisation and perpetration. Participants were assigned to the closest trajectory based on the data available, with missing data handled by the *CopyMean* imputation method for participants with data for at least one time point. *CopyMean* first uses the Last Occurrence Carried Forward (LOCF) method to obtain an approximation of the imputed value, and then uses the population mean trajectory to refine the first approximation. *KmL3D* ran 500 times with different initial assignments to find the best two-cluster solution. The whole process was repeated with the number of clusters (i.e., trajectories) ranging from 2 to 6, to identify the optimal number of trajectories. We selected the best solution based on several model-fitting criteria (see Appendix S1 for details). As sensitivity analyses, we re-estimated the trajectories using (1) reports from single informants (i.e., parent reports and child reports only) and (2) participants with data from at least two time points (instead of one time point).

#### Early risk factors for joint trajectories of bullying victimisation and perpetration

To test associations between child and family factors with trajectories of bullying involvement, we used multinomial logistic regressions with the *nnet* package [34]. First, we ran univariate regression models to assess the association between each early risk factor and bullying trajectory separately. Next, we ran multivariate regression models to assess if each risk factor was independently associated with each trajectory. Finally, we repeated analyses contrasting the bully-victim group with “pure” victim and bully trajectories, to identify potentially distinct risk factors for bully-victims. For all regression models except for the bully-victim analysis, the uninvolved trajectory (children with the lowest levels of victimisation and perpetration) was used as the reference group. We did not compare the relative strength of effect sizes across early risk factors as it was not possible to standardise all early risk factors (e.g., binary variables like male sex or physical health problems).

#### Multiple imputation

Missing data in the early risk factors were imputed using multiple imputation by chained equations (*n* = 50 datasets with 30 iterations) with the *mice* package [35]. Proportions of missing data for all variables are shown in Table S6. Results from the complete case sample (minimum *N* = 11,338) are presented in the supplementary materials.

## Results

### Joint developmental trajectories of bullying victimisation and perpetration

**Fig. 1:**
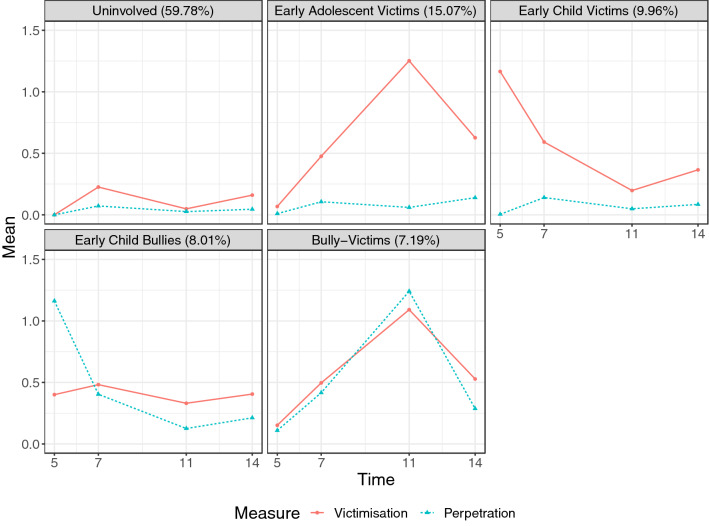
The five joint trajectories of bullying victimisation and perpetration from childhood to adolescence

We identified five joint trajectories of bullying involvement from childhood (age 5) to adolescence (age 14): uninvolved children, early child victims, early adolescent victims, early child bullies, and bully victims (Fig. 1, Appendix S1, Tables S7–S8, Figures S1–S3). The uninvolved trajectory (*n* = 8,706 [59.78%]) included children who had zero or close to zero ratings at all time points (ages 5, 7, 11, 14). The early child victim trajectory (*n* = 1,450 [9.96%]) included children with high levels of victimisation at age 5 which decreased across ages 7 and 11 and remained low at age 14, and no perpetration. The early adolescent victim trajectory (*n* = 2,195 [15.07%]) included individuals who had low increasing victimisation in childhood which peaked at age 11 and decreased to moderate victimisation at age 14, and no perpetration. The early child bully trajectory (*n* = 1,166 [8.01%]) included children with high perpetration at age 5 which decreased over time, and low victimisation across all ages. The bully-victim trajectory (*n* = 1,047 [7.19%]) included children who had simultaneously increasing, then decreasing levels of victimisation and perpetration from childhood to adolescence (low at age 5, increasing to moderate at age 7 and high at age 11, then decreasing again to low at age 14).

#### Sensitivity analyses

Trajectories estimated from parent reports only (at ages 5, 7, and 14) were similar to those estimated from multiple informants, identifying groups of uninvolved children, early child victims, early adolescent victims, early child bullies, and bully victims (Figure S4, Tables S15–S16). Trajectories estimated from child reports only (at ages 7, 11, and 14) were similar but not identical to multi-informant trajectories (likely because of the difference in observational period), and included groups of uninvolved children, child victims, chronic victims (with peak in early adolescence), child bully victims, and adolescent bully victims (Figure S5, Tables S17–S18). The results remained similar when trajectories were estimated from participants with available data from multiple time points (Figure S6).

### Early risk factors for joint trajectories of bullying victimisation and perpetration

The distribution of early risk factors across joint trajectories of victimisation and perpetration is presented in Table S9.

Univariate analyses revealed that a range of early child and family factors were associated with trajectories of bullying involvement (Table S10). Findings from multivariate analyses testing the independent associations between child and family risk factors and trajectories of bullying involvement are presented in Table 1 and discussed below.

**Table 1:** Multivariate regression for independent associations between early child and family factors with bullying involvement trajectories

Early factor	Early child victims		Early adolescent victims		Early child bullies		Bully-victims	
	OR	95% CI	OR	95% CI	OR	95% CI	OR	95% CI
*Child*								
Male sex	1.26	1.15–1.38***	1.10	1.00–1.19*	1.82	1.68–1.96***	1.90	1.76–2.04***
Birthweight	0.93	0.83–1.03	0.90	0.82–0.99*	0.90	0.79–1.01	0.99	0.88–1.11
BMI	1.04	1.01–1.06**	1.04	1.02–1.06***	1.03	1.00–1.05*	1.03	1.00–1.06*
Physical health problem	1.60	1.48–1.73***	1.16	1.05–1.26**	1.24	1.09–1.38**	1.06	0.91–1.21
Positive mood as an infant	1.02	0.96–1.08	1.01	0.96–1.06	0.98	0.92–1.05	1.03	0.96–1.10
Withdrawn as an infant	1.00	0.93–1.06	0.92	0.86–0.97**	1.00	0.92–1.07	0.95	0.88–1.03
Unadaptable as an infant	1.03	0.96–1.09	0.95	0.89–1.00*	0.97	0.89–1.04	0.93	0.85–1.00*
Regular eating/sleeping as an infant	0.93	0.87–0.99*	1.00	0.95–1.06	0.94	0.87–1.01	0.95	0.89–1.02
Independence and self-regulation	0.95	0.89–1.02	0.94	0.88–0.99*	0.92	0.85–0.99*	0.93	0.86–1.00*
Emotional dysregulation	1.23	1.16–1.29***	1.12	1.07–1.18***	1.71	1.64–1.79***	1.21	1.13–1.29***
Cognitive ability	0.88	0.81–0.94***	0.98	0.93–1.04	0.85	0.78–0.92***	0.81	0.74–0.88***
*Family*								
Positive mother–child relationship	0.98	0.91–1.05	1.00	0.94–1.07	0.82	0.74–0.90***	0.93	0.84–1.01
Harsh maternal discipline	1.03	0.97–1.09	1.13	1.08–1.19***	1.45	1.38–1.52***	1.24	1.17–1.31***
Maternal mental health problems	1.36	1.30–1.41***	1.09	1.03–1.14**	1.19	1.12–1.25***	1.10	1.03–1.17**
Low family income	1.19	1.14–1.23***	1.03	0.99–1.06	1.33	1.28–1.39***	1.25	1.20–1.31***
Family size	0.94	0.90–0.99*	0.95	0.91–1.00*	1.05	1.00–1.10*	1.03	0.98–1.08

Measures of infant temperament, independence and self-regulation, emotional dysregulation, cognitive ability, mother–child relationship, maternal discipline, and maternal mental health were standardised

*OR* odds ratio, *CI* confidence interval, BMI body mass index

*p < .05; **p < .01; ***p < .001 with uninvolved children as the reference group

#### Child factors

Certain child vulnerabilities were independently associated with increased risk for all trajectories of bullying involvement. For example, boys and children with higher early BMI and emotional dysregulation in the preschool years were more likely to become early child victims, early adolescent victims, early child bullies, and bully-victims. Furthermore, early cognitive difficulties, physical health problems, and low independence and self-regulation in infancy were also risk factors for multiple trajectories of bullying involvement. We also observed distinct risk factors for specific trajectories: for example, children with low birthweight were at an increased risk for becoming early adolescent victims.

#### Family factors

Various family factors independently forecasted multiple trajectories of bullying involvement. For example, poor maternal mental health in early life was uniquely associated with all trajectories of bullying involvement, while harsh maternal discipline and low income forecasted becoming a bully victim, early child bully, and victim in adolescence or early childhood, respectively. Risk factors for specific trajectories also emerged: for example, preschool children who had poor relationships with their mothers were more likely to become early child bullies. Furthermore, coming from a large family was associated with a lower risk of becoming an early child victim and early adolescent victim, but a higher  risk of being an early child bully. Results were broadly consistent for the complete case sample (Tables S11–S12).

### Distinct risk factors for becoming a bully-victim

Bully victims differed from early child and adolescent victims (hereafter “victims”) and early child bullies in various early child and family factors (Table S13). Compared to victims, bully victims were more likely to be boys and have lower cognitive ability. Bully victims also experienced greater environmental adversity than victims, with harsher maternal discipline, lower family income, and larger family sizes. However, early child bullies showed more early vulnerabilities than bully victims, with worse emotional dysregulation, harsher maternal discipline, and less positive maternal relationships. Results were broadly consistent for the complete case sample (Table S14).

## Discussion

To our knowledge, this is the first study to (1) describe joint trajectories of bullying victimisation and perpetration from early childhood to adolescence, and (2) identify early risk factors for these trajectories. Using data from a large, contemporary UK birth cohort, the analyses revealed three key findings.

First, we identified five joint trajectories of bullying victimisation and perpetration from early childhood to adolescence: children who were uninvolved (59.78%), early child victims (9.96%), early adolescent victims (15.07%), early child bullies (8.01%), and bully victims (7.19%). Notably, distinct groups of children experienced victimisation in early childhood versus in early adolescence, with no perpetration. This supports findings from previous studies which found distinct trajectories of decreasing and increasing victimisation, with minimal or no perpetration [12, 15]. While we did not find a group of children who were chronically victimised across the full 9-year period from ages 5 to 14, bully victims and early adolescent victims experienced elevated levels of victimisation between ages 7 to 14 years, which peaked at age 11. Therefore, it is possible that previous studies identifying groups of chronically victimised children [5, 11, 18, 25] may partly be capturing bully-victims, as well as “pure” victims. Another striking finding is that there were no “pure” bullies in early adolescence, only bully victims. This supports findings from contemporary cohorts from Australia [15], China [18], and South Korea [16, 17], and suggests that adolescents who bully others are also likely to be victims of bullying. Notably, these findings were largely consistent when trajectories were estimated with reports from single informants (parents or children only) instead of multiple informants. Overall, the results highlight the importance of mapping joint trajectories of victimisation and perpetration, to provide insight into the heterogeneous developmental patterns of bullying involvement.

Second, we found that a host of early child and familial vulnerabilities independently forecasted multiple trajectories of bullying involvement. Regarding child factors, emotional and cognitive vulnerabilities (emotional dysregulation, cognitive difficulties, and low infant independence and self-regulation) and physical vulnerabilities (high BMI and physical health problems) in pre-school years were associated with becoming a bully victim, early child bully, and victim (in childhood and/or adolescence). Boys were also more likely than girls to become involved in all trajectories of bullying involvement. Regarding family vulnerabilities, poor maternal mental health, harsh maternal discipline and low family income were associated with becoming a bully victim, early child bully, and victim (in childhood and adolescence). These findings extend research linking these risk factors to bullying involvement primarily in cross-sectional studies [20, 26] and longitudinal studies of victimisation or perpetration only [6, 24, 25, 36]. However, this is the first study to identify these early predictors of joint trajectories of bullying victimisation and perpetration. Given that these early factors were associated with multiple trajectories of bullying involvement, interventions targeting these risk factors may be particularly beneficial in preventing bullying.

Third, we found that bully-victims differed in early risk factors to victims and early child bullies. Bully-victims exhibited higher levels of early risk factors than victims: for example, they were more likely to be male, have lower cognitive ability, experience harsher maternal discipline and lower family income, and come from larger families. Surprisingly though, early child bullies showed higher levels of early risk factors than bully-victims, with more emotional dysregulation, worse mother–child relationships, and harsher maternal discipline. This finding counters prior research using researcher-defined categories of bullies or bully victims, which has suggested that bully victims have higher levels of risk factors than “pure” bullies [13, 14]. This may reflect a closer temporal relationship between the measurement of early risk factors (between infancy and age 5) and involvement in bullying, as early child bullies showed the highest levels of bullying at age 5, while bully-victims showed low levels of bullying involvement at age 5 and 7, and higher levels at age 11 and 14. Alternatively, it may reflect reporting bias, as bullying involvement at age 5 was reported only by mothers, and mothers who reported more difficulties with their children may also be more likely to report more bullying by their child. Therefore, it will be important to test whether this finding replicates in other longitudinal cohorts with data on bullying from multiple informants.

These findings should be considered in the context of some limitations. First, many of the items used to measure bullying in the MCS did not assess key elements of the research definition (i.e., intentionality, repetition, and power differential between bully and victim) [37], but instead relied on parents’, teachers’, and children’s personal definitions of “bullying”. As such, the bullying construct measured likely reflects parents’, teachers’, and children’s conceptions of bullying, which may not fully overlap with the formal research definition [38, 39]. However, we note that these measures are comparable to bullying measures in other longitudinal studies [11, 36, 40] which do not directly assess elements of the research definition. Second, bullying victimisation and perpetration were assessed via single items (respectively) for each informant, at each time point. Third, the measures of bullying at age 5 were reported only by mothers, and not multiple informants (as at the later time points). However, parents have been shown to be reliable informants of their children’s bullying involvement, particularly in early childhood [28]. Fourth, in the multivariate analysis, it was not possible to determine whether attenuation in the effects of some risk factors reflected confounding or mediation by other covariates. For example, the null associations between birthweight and the early child victim and child bully trajectories in the multivariate analyses might have emerged because we controlled for potential mediators (e.g., cognitive ability) on the causal pathway. Lastly, although risk factors were assessed prior to bullying, we cannot infer that risk factors caused involvement in bullying as there may be unmeasured confounding (e.g., from genetic influences). Future studies using genetically informed causal inference methods [41–43] are required to test the causal effect of risk factors on bullying involvement.

Despite these limitations, our findings have implications for future research and interventions. With regard to research, our findings highlight the importance of also considering bullying perpetration in research on victimisation. We found that around 1 in 5 victimised children also bullied others, and these bully-victims had chronic exposure to victimisation and higher levels of early risk factors than “pure” victims. Therefore, researchers studying the consequences of victimisation should test (1) whether poor outcomes are present in both victims and bully-victims, and (2) if potentially worse outcomes in bully-victims [7] partly reflect confounding by higher levels of early child and family vulnerabilities.

Pending further studies confirming the causal effect of the identified risk factors, interventions addressing these risk factors could help prevent children from becoming involved in trajectories of bullying involvement. Regarding child vulnerabilities, interventions could include early mental health care or skills-based training to improve emotional regulation [44], which could help to prevent other long-term adverse outcomes linked to early vulnerabilities (e.g., health problems and socioeconomic outcomes) [45] as well as bullying. Other school-based interventions which aim to reduce stigma towards some of the child risk factors we identified (e.g., obesity or cognitive impairments) [46] could also help prevent bullying. Regarding family vulnerabilities, antipoverty programs, parenting support schemes, and more accessible mental health care could improve parent wellbeing and child–parent relationships [47, 48] and reduce risk of subsequent bullying involvement. Furthermore, although bullying victimisation contributes independently to the development of mental health problems [3], the risk factors that we identified for bullying also increase risk for mental health problems more broadly [49]. Thus, addressing these early risk factors could not only protect children from bullying, but also reduce their risk of developing impairing mental health problems.

## Supplementary Information

Below is the link to the electronic supplementary material.

Supplementary file1 (DOCX 2073 KB)
