# Supplementary material for: Early risk factors for joint trajectories of bullying victimisation and perpetration
Source: Eur Child Adolesc Psychiatry. 2022 Apr 25;32(9):1723–31. doi: 10.1007/s00787-022-01989-6 (PMC10460348; doi:10.1007/s00787-022-01989-6)
Supplement: Supplementary file 1 — Supplementary file1 (DOCX 2073 KB) [file 787_2022_1989_MOESM1_ESM.docx]

Supplementary Materials

Early risk factors for joint trajectories of bullying victimisation and perpetration

European Child & Adolescent Psychiatry

Athena R.W. Chow, BSc; Jean-Baptiste Pingault, PhD; Jessie R. Baldwin, PhD

Table of Contents

[Appendix S1. Rationale for selecting five-trajectory solution. 3](#_Toc99036576)

[Table S1. STROBE (Strengthening the Reporting of Observational studies with Epidemiology) Checklist of items that should be included in reports of cohort studies. 5](#_Toc99036577)

[Table S2. Bullying victimisation and perpetration measures from multiple informants for ages 5, 7, 11, and 14. 7](#_Toc99036578)

[Table S3. Correlation matrix of victimisation measures across Sweeps 3, 4, 5, 6 (ages 5, 7, 11, 14). 9](#_Toc99036579)

[Table S4. Correlation matrix of perpetration measures across Sweeps 3, 4, 5, 6 (ages 5, 7, 11, 14). 9](#_Toc99036580)

[Table S5. Details on how each early factor was documented and derived. 10](#_Toc99036581)

[Table S6. Missing data in study variables before multiple imputation. 13](#_Toc99036582)

[Table S7. Indices to determine the best-fitting model between 2-6 latent clusters. 14](#_Toc99036583)

[Table S8. Posterior probabilities of each trajectory. 14](#_Toc99036584)

[Table S9. Distribution of sociodemographic characteristics and early risk factors across joint trajectories of bullying victimisation and perpetration from multiply imputed data. 15](#_Toc99036585)

[Table S10. Imputed data univariate regressions for associations between early child and family factors with bullying involvement trajectories. 16](#_Toc99036586)

[Table S11. Complete data univariate regressions for associations between early child and family factors with bullying involvement trajectories. 17](#_Toc99036587)

[Table S12. Complete data multivariate regression for independent associations between early child and family factors with bullying involvement trajectories. 18](#_Toc99036588)

[Table S13. Multivariate regression with bully-victims coded as the reference group, conducted on multiply imputed data. 19](#_Toc99036589)

[Table S14. Multivariate regression with bully-victims coded as the reference group, conducted on complete cases. 20](#_Toc99036590)

[Figure S1. The five joint trajectories of bullying victimisation and perpetration. 21](#_Toc99036591)

[Figure S2. Three-dimensional graphs of mean joint trajectories with time on the x-axis, perpetration on the y-axis, and victimisation on the z-axis. 22](#_Toc99036592)

[Figure S3. Six-trajectory solution of the joint trajectories of victimisation and perpetration. 23](#_Toc99036593)

[Table S15. Posterior probabilities for parent-reported trajectory solutions. 24](#_Toc99036594)

[Table S16. Model-fitting indices for parent-reported trajectory solutions. 25](#_Toc99036595)

[Table S17. Posterior probabilities for child-reported trajectory solutions. 25](#_Toc99036596)

[Table S18. Model-fitting indices for child-reported trajectory solutions. 26](#_Toc99036597)

[Figure S4. Parent-reported five-trajectory solution. 27](#_Toc99036598)

[Figure S5. Child-reported five-trajectory solution. 27](#_Toc99036599)

[Figure S6. Multi-informant five-trajectory solution, when we used a different missing data requirement (maxNA=2). 29](#_Toc99036601)

# Appendix S1. Rationale for selecting five-trajectory solution.

We selected the five-trajectory solution based on model-fitting criteria and previous evidence on joint trajectories of bullying victimisation and perpetration.

*Model-fitting criteria*. To identify the optimal number of clusters, *KmL3D* computes several fit indices: the Calinski-Harabasz (Caliński & Harabasz, 1974), Davies-Bouldin (Davies & Bouldin, 1979), Ray-Turi (Ray & Turi, 1999), Akaike information criterion (AIC; Akaike, 1974), and Bayesian information criterion (BIC; Schwarz, 1978). In the context of trajectories, posterior probabilities (Bolstad, 2007) reflect the probability that participants in a given trajectory actually belong to this trajectory, so that a probability is given for each trajectory. Criteria tend to choose extreme solutions (e.g., very high or very low) and accordingly, the optimal number of trajectories was either six (Calinski-Harabasz, BIC, and AIC) or two (Ray-Turi, Davies-Bouldin) (Table S7). Notably though, the five-trajectory solution was identified as the joint best by the Davies-Bouldin criterion and as the second-best by the Calinski-Harabasz, BIC, and AIC indices. Posterior probabilities were high for the two and five-trajectory solutions (i.e., above 0.70), but one was inadequate for the six-trajectory solution (0.68) (Table S8).

*Previous evidence.* We considered the five, two, and six trajectory solutions in the context of previous evidence (van Lier et al., 2007). The five-trajectory solution (Figures 1, S1, and S2) was most consistent with previous evidence as it included an uninvolved subgroup, one bully subgroup, two victim subgroups, and one bully-victim subgroup (Barker et al., 2008; Haltigan & Vaillancourt, 2014; Zhou et al., 2020). The two-trajectory solution was not consistent with previous evidence because it only included an uninvolved group and an involved group. The six-trajectory solution included an uninvolved group, three victimisation trajectories (early child victims, early adolescent victims, mid-adolescent victims), one child bully trajectory, and one bully-victim trajectory (see Figure S3). The six-trajectory solution did not align with previous research which found six joint trajectories of bullying as there was no victim-to-bully subgroup (Barker et al., 2008) nor was there a moderate/stable bully-victim subgroup (Barker et al., 2008; Walters, 2021). Because the five-trajectory solution was most consistent with prior studies modelling joint trajectories of victimisation and perpetration, and had the highest or second highest scores on model-fitting indices, as well as appropriate posterior probabilities for each trajectory, we selected the five-trajectory solution.

# Table S1. STROBE (Strengthening the Reporting of Observational studies with Epidemiology) Checklist of items that should be included in reports of cohort studies.

|  | Item | Recommendation | Page |
| --- | --- | --- | --- |
| **Title and abstract** | 1 | (*a*) Indicate the study’s design with a commonly used term in the title or the abstract | 1-2 |
|  |  | (*b*) Provide in the abstract an informative and balanced summary of what was done and what was found |  |
| Introduction | | | |
| Background/rationale | 2 | Explain the scientific background and rationale for the investigation being reported | 3-4 |
| Objectives | 3 | State specific objectives, including any prespecified hypotheses | 4-5 |
| Methods | | | |
| Study design | 4 | Present key elements of study design early in the paper | 5-6 |
| Setting | 5 | Describe the setting, locations, and relevant dates, including periods of recruitment, exposure, follow-up, and data collection | 5-6 |
| Participants | 6 | (*a*) Give the eligibility criteria, and the sources and methods of selection of participants. Describe methods of follow-up | 5-6 |
|  |  | (*b*) For matched studies, give matching criteria and number of exposed and unexposed |  |
| Variables | 7 | Clearly define all outcomes, exposures, predictors, potential confounders, and effect modifiers. Give diagnostic criteria, if applicable | 6-7, S2, S5 |
| Data sources/ measurement | 8* | For each variable of interest, give sources of data and details of methods of assessment (measurement). Describe comparability of assessment methods if there is more than one group | S2, S5 |
| Bias | 9 | Describe any efforts to address potential sources of bias | 7-8 |
| Study size | 10 | Explain how the study size was arrived at | 7-8 |
| Quantitative variables | 11 | Explain how quantitative variables were handled in the analyses. If applicable, describe which groupings were chosen and why | S2, S5 |
| Statistical methods | 12 | (*a*) Describe all statistical methods, including those used to control for confounding | 7-8 |
|  |  | (*b*) Describe any methods used to examine subgroups and interactions |  |
|  |  | (*c*) Explain how missing data were addressed |  |
|  |  | (*d*) If applicable, explain how loss to follow-up was addressed |  |
|  |  | (*e*) Describe any sensitivity analyses |  |
| Results | | |  |
| Participants | 13* | (a) Report numbers of individuals at each stage of study—eg numbers potentially eligible, examined for eligibility, confirmed eligible, included in the study, completing follow-up, and analyzed | 8-9, S9, S11 |
|  |  | (b) Give reasons for non-participation at each stage |  |
|  |  | (c) Consider use of a flow diagram |  |
| Descriptive data | 14* | (a) Give characteristics of study participants (eg demographic, clinical, social) and information on exposures and potential confounders | S6, S9 |
|  |  | (b) Indicate number of participants with missing data for each variable of interest |  |
|  |  | (c) Summarize follow-up time (eg, average and total amount) |  |
| Outcome data | 15* | Report numbers of outcome events or summary measures over time | 9-11 |
| Main results | 16 | (*a*) Give unadjusted estimates and, if applicable, confounder-adjusted estimates and their precision (eg, 95% confidence interval). Make clear which confounders were adjusted for and why they were included  (*b*) Report category boundaries when continuous variables were categorized  (*c*) If relevant, consider translating estimates of relative risk into absolute risk for a meaningful time period | Table 1,  S10, S11, S12, S13, S14 |

| Other analyses | 17 | Report other analyses done—eg analyses of subgroups and interactions, and sensitivity analyses | 9-11, S13, S14, S15, S16, S17, S18 |
| --- | --- | --- | --- |
| Discussion | | | |
| Key results | 18 | Summarize key results with reference to study objectives | 11-13 |
| Limitations | 19 | Discuss limitations of the study, taking into account sources of potential bias or imprecision. Discuss both direction and magnitude of any potential bias | 13-14 |
| Interpretation | 20 | Give a cautious overall interpretation of results considering objectives, limitations, multiplicity of analyses, results from similar studies, and other relevant evidence | 11-15 |
| Generalizability | 21 | Discuss the generalizability (external validity) of the study results | 14-15 |
| Other information | | | |
| Funding | 22 | Give the source of funding and the role of the funders for the present study and, if applicable, for the original study on which the present article is based | 1 |

*Note.* Page numbers refer to pages in the main text, but ‘S’ refers to items in this supplement.

# Table S2. Bullying victimisation and perpetration measures from multiple informants for ages 5, 7, 11, and 14.

***Bullying victimisation measures***

| **Age** | **Informant** | **Question** | **Responses** | **Coding** | **Mean (SD)** | **Composite Mean (SD)** |
| --- | --- | --- | --- | --- | --- | --- |
| 5 | Parent | Picked on or bullied by other children | “not true”,  “somewhat true”,  “certainly true” | 0 = “not true”,  1= “somewhat true”  2= certainly true” | 0.16 (0.42) | 0.16 (0.42) |
| 7 | Parent | Picked on or bullied by other children | “not true”,  “somewhat true”,  “certainly true” | 0 = “not true”,  1= “somewhat true”  2= certainly true” | 0.24 (0.50) | 0.34 (0.41) |
|  | Teacher | Picked on or bullied by other children | “not true”,  “somewhat true”,  “certainly true” | 0 = “not true”,  1= “somewhat true”  2= certainly true” | 0.09 (0.35) |  |
|  | Child | How often do other children bully you? | “Never”  “Some of the time”  “All of the time” | 0 = “Never”  1 = “Some of the time”  2 = “All of the time” | 0.58 (0.65) |  |
| 11 | Teacher | Picked on or bullied by other children | “not true”,  “somewhat true”,  “certainly true” | 0 = “not true”,  1= “somewhat true”  2= certainly true” | 0.14 (0.40) | 0.35 (0.59) |
|  | Child | How often do other children hurt you or pick on you on purpose? | “Never”  “Less often”  “Every few months”  “About once a month”  “About once a week”  “Most days” | 0 = “Never” \| “Less often”  1 = “Every few months” \| “About once a month”  2- “About once a week” \| “Most days” | 0.44 (0.74) |  |
| 14 | Parent | Picked on or bullied by other children | “not true”,  “somewhat true”,  “certainly true” | 0 = “not true”,  1= “somewhat true”  2= certainly true” | 0.28 (0.56) | 0.30 (0.49) |
|  | Child | How often do other children hurt you or pick on you on purpose? | “Never”  “Less often”  “Every few months”  “About once a month”  “About once a week”  “Most days” | 0 = “Never” \| “Less often”  1 = “Every few months” \| “About once a month”  2- “About once a week” \| “Most days” | 0.31 (0.65) |  |

***Bullying perpetration measures***

| **Age** | **Informant** | **Question** | **Responses** | **Coding** | **Mean (SD)** | **Composite Mean (SD)** |
| --- | --- | --- | --- | --- | --- | --- |
| 5 | Parent | Fights with or bullies other children | “not true”,  “somewhat true”,  “certainly true” | 0 = “not true”,  1= “somewhat true”  2= certainly true” | 0.10 (0.34) | 0.10 (0.34) |
| 7 | Parent | Fights with or bullies other children | “not true”,  “somewhat true”,  “certainly true” | 0 = “not true”,  1= “somewhat true”  2= certainly true” | 0.08 (0.32) | 0.14 (0.29) |
|  | Teacher | Fights with or bullies other children | “not true”,  “somewhat true”,  “certainly true” | 0 = “not true”,  1= “somewhat true”  2= certainly true” | 0.15 (0.42) |  |
|  | Child | How often are you horrible to other children at school? | “Never”  “Some of the time”  “All of the time” | 0 = “Never”  1 = “Some of the time”  2 = “All of the time” | 0.17 (0.44) |  |
| 11 | Teacher | Fights with or bullies other children | “not true”,  “somewhat true”,  “certainly true” | 0 = “not true”,  1= “somewhat true”  2= certainly true” | 0.14 (0.39) | 0.12 (0.35) |
|  | Child | How often do you hurt or pick on other children on purpose? | “Never”  “Less often”  “Every few months”  “About once a month”  “About once a week”  “Most days” | 0 = “Never” \| “Less often”  1 = “Every few months” \| “About once a month”  2- “About once a week” \| “Most days” | 0.12 (0.40) |  |
| 14 | Parent | Fights with or bullies other children | “not true”,  “somewhat true”,  “certainly true” | 0 = “not true”,  1= “somewhat true”  2= certainly true” | 0.08 (0.33) | 0.09 (0.28) |
|  | Child | How often do you hurt or pick on other children on purpose? | “Never”  “Less often”  “Every few months”  “About once a month”  “About once a week”  “Most days” | 0 = “Never” \| “Less often”  1 = “Every few months” \| “About once a month”  2- “About once a week” \| “Most days” | 0.10 (0.37) |  |

# Table S3. Correlation matrix of victimisation measures across Sweeps 3, 4, 5, 6 (ages 5, 7, 11, 14).


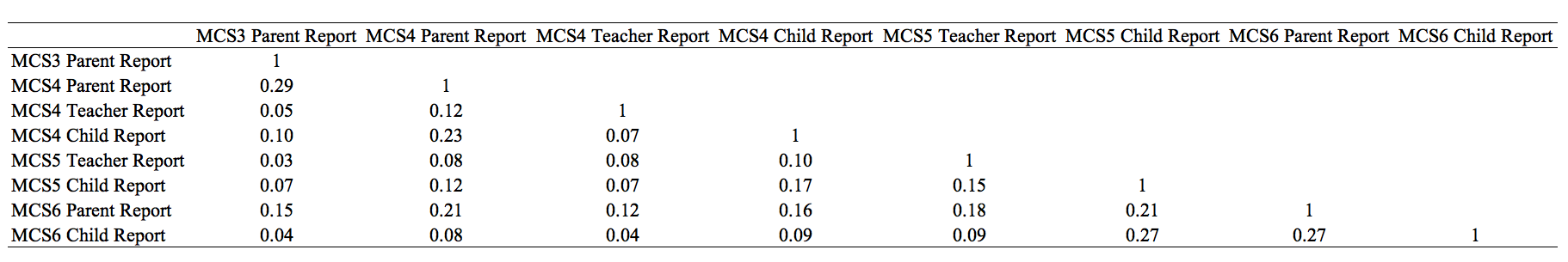


# Table S4. Correlation matrix of perpetration measures across Sweeps 3, 4, 5, 6 (ages 5, 7, 11, 14).


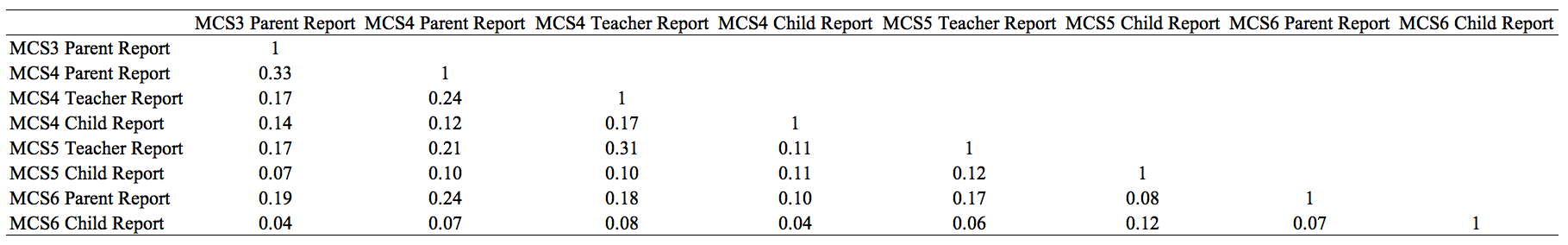


# Table S5. Details on how each early factor was documented and derived.

| **Early Factor** | **Age(s) Assessed** | **Informant** | **Description** |
| --- | --- | --- | --- |
| ***Child*** | | | |
| Sex | 9 months | Parent | Biological sex was documented as male or female. |
| Birthweight | 9 months | Parent | Birthweight was documented in grams *(g)*. |
| Body Mass Index (BMI) | 3 & 5 years | MCS observer | BMI *(kg/m^2^)* was recorded at sweeps 2 and 3, which was averaged to form a measure of early BMI. |
| Physical health problem | 3 & 5 years | Mother | Mothers were asked whether the child has “any long-standing limiting illness lasting over 12 months which limits the child in their daily activities” at sweeps 2 and 3. Both measures were combined and coded to be 0 (no illness at either time point) or 1 (having an illness at one or both time points). Examples of commonly reported illnesses included asthma, dermatitis, and traumatic pneumothorax. |
| Infant mood | 9 months | Mother | Infant mood was measured at sweep 1 with the Carey Infant Temperament Scale.(Carey & McDevitt, 1978) Mothers rated how often their infant’s temperament matched 5 mood items (e.g. “happy sounds during nappy change”, “pleasant when arriving in unfamiliar places”, “pleasant during hair brushing”, “content during interruptions of feeding”, and “pleasant or calm with minor injuries”). Scores were summed across 5 items to form a measure of infant mood (with higher scores indicating more positive mood), then standardised. |
| Infant approach /withdrawal | 9 months | Mother | Infant approach and withdrawal was measured at sweep 1 with the Carey Infant Temperament Scale.(Carey & McDevitt, 1978) Mothers rated how often their infant’s temperament matched 3 approach/withdrawal items (e.g. “objects to being bathed in a different place or by a different person”, “still wary of strangers after 15 minutes”, and “shy on meeting another child for the first time”). Scores were summed across 3 items to form a measure of infant approach/withdrawal (with higher scores indicating more withdrawn infants), then standardised. |
| Infant adaptability | 9 months | Mother | Infant adaptability was measured at sweep 1 with the Carey Infant Temperament Scale.(Carey & McDevitt, 1978) Mothers rated how often their infant’s temperament matched 3 adaptability items (e.g. “fretful for the first few minutes in a new place”, “bothered when put down in different sleeping place”, and “wants/takes milk at about the same time from day to day”). Scores were summed across 3 items to form a measure of infant adaptability (with higher scores indicating lower adaptability), then standardised. |
| Infant regularity | 9 months | Mother | Infant regularity was measured at sweep 1 with the Carey Infant Temperament Scale.(Carey & McDevitt, 1978) Mothers rated how often their infant’s temperament matched 3 regularity items (e.g. “gets sleepy at about the same time”, “naps for the same length of time”, and “wants and takes solid food at about the same time”). Scores were summed across 3 items to form a measure of infant regularity (with higher scores indicating higher regularity), then standardised. |
| Independence and self-regulation | 3 & 5 years | Mother | Independence and self-regulation were measured at sweeps 2 and 3 using the Child Social Behavior Questionnaire.(Hogan et al., 1992) Mothers rated how true their child’s behavior reflected 5 independence and self-regulation items (e.g. “likes to work things out for self”, “does not need much help with tasks”, “chooses activities on their own”, “persists in the face of difficult tasks”, and “move to new activity after finishing task”). Scores were derived by summing the mean of responses for each domain (with higher scores indicating higher independence and self-regulation), then standardised. |
| Emotional dysregulation | 3 & 5 years | Mother | Emotional dysregulation was measured at sweeps 2 and 3 using the Child Social Behavior Questionnaire.(Hogan et al., 1992) Mothers rated how true their child’s behavior reflected 5 emotional dysregulation items (e.g. “shows mood swings”, “gets over excited”, “easily frustrated”, “gets over being upset slowly”, and “acts impulsively”). Scores were derived by summing the mean of responses for each domain (with higher scores indicating higher emotional dysregulation), then standardised. |
| Cognitive ability | 5 years | Child assessment | Cognitive ability was assessed at sweep 3 using 3 test scales of the British Ability Scales (BAS)(Elliott et al., 1996): Naming Vocabulary (this measured expressive language ability as children were asked to name colored pictures of objects), Pattern Construction (this measured spatial awareness as children had to construct different patterns by putting together flat squares or solid cubes), and Picture Similarities (this measured problem solving abilities as children were asked to choose the most similar picture after being shown a row of examples). T-scores (adjusted for the child’s age group and for the mean scores of the BAS norming group) for each ability were summed to form an overall cognitive ability score (with higher scores indicating higher cognitive ability), then standardised. |
| ***Family*** | | | |
| Mother-child relationship | 3 years | Mother | Mother-child relationship was assessed at sweep 2 using the short form Child-Parent Relationship Scale.(Pianta, 1992) The Pianta scale, consisting of 15 items, assessed the mother-child relationship between mother and cohort member (CM) (e.g. “I share an affectionate relationship with CM”, “CM will seek comfort from me”, “CM values his/her relationship with me”, “CM spontaneously shares information about himself/herself”, and “it is easy to be in tune with what CM is feeling”). Scores were summed to form a measure of overall positive mother-child relationship (with higher scores indicating a more positive relationship), then standardised. |
| Maternal discipline style | 3 & 5 years | Mother | Discipline style was documented at sweeps 2 and 3 using Straus’s Conflict Tactics Scale.(Straus & Hamby, 1997) Mothers rated across 6 items how often they practiced the discipline method when the CM was naughty (e.g. “ignore CM”, “smack CM”, “shout at CM”, “send CM to his/her bedroom/naughty chair”, “take away treats”, and “tell CM off”). Maternal discipline scores were summed and combined across both sweeps (with higher scores indicating harsher discipline), then standardised. |
| Maternal mental health | 3 & 5 years | Mother | The Kessler (K6) scale,(Kessler et al., 2003) consisting of 6 items, was administered to mothers at sweeps 2 and 3. Mothers were asked how often in the last 30 days they felt depressive and anxiety-related symptoms (e.g. “so depressed that nothing could cheer you up”, “hopeless”, “restless or fidgety”, “that everything was an effort”, “worthless”, and “nervous”). Mental health scores were summed and combined across both sweeps (with higher scores indicating worse mental health), then standardised. |
| Family income quintile | 9 months | Parent | Family income was documented at sweep 1. Equivalized household income was weighted for the number of adults and dependent children in the household, divided into five quintiles (lowest, second, third, fourth, and highest). Family income scores were reverse-coded such that a higher score reflected poverty. |
| Family size | 3 years | Parent | Family size was documented at sweep 2, measured as number of people in the household including the child. |

# Table S6. Missing data in study variables before multiple imputation.

| **Variable** | **N** | **Missing N** | **Proportion missing** |
| --- | --- | --- | --- |
| **Child risk factor** |  |  |  |
| Sex | 18,552 | 0 | 0 |
| Birthweight | 18,487 | 65 | 0.004 |
| BMI | 16,159 | 2,393 | 0.129 |
| Physical health problem | 13,672 | 4,880 | 0.263 |
| Infant mood | 17,919 | 633 | 0.034 |
| Infant approach/withdrawal | 17,880 | 672 | 0.036 |
| Infant adaptability | 17,911 | 641 | 0.035 |
| Infant regularity | 17,910 | 642 | 0.035 |
| Independence and self-regulation | 15,999 | 2,553 | 0.138 |
| Emotional dysregulation | 15,999 | 2,553 | 0.138 |
| Cognitive ability | 14,441 | 4,111 | 0.222 |
| **Family risk factor** |  |  |  |
| Mother-child relationship | 13,123 | 5,429 | 0.293 |
| Maternal discipline style | 15,451 | 3,101 | 0.167 |
| Maternal mental health | 15,410 | 3,142 | 0.169 |
| Family income quintile | 18,470 | 82 | 0.004 |
| Family size | 14,898 | 3,654 | 0.197 |
| **Bullying measures** |  |  |  |
| Victimisation at age 5 | 14,108 | 1,138 | 0.075 |
| Victimisation at age 7 | 13,127 | 2,119 | 0.139 |
| Victimisation at age 11 | 12,165 | 3,081 | 0.202 |
| Victimisation at age 14 | 10,976 | 4,270 | 0.280 |
| Perpetration at age 5 | 14,606 | 640 | 0.042 |
| Perpetration at age 7 | 13,142 | 2,104 | 0.138 |
| Perpetration at age 11 | 12,170 | 3,076 | 0.202 |
| Perpetration at age 14 | 10,980 | 4,266 | 0.280 |

# Table S7. Indices to determine the best-fitting model between 2-6 latent clusters.

| No. Clusters | Calinski-Harabasz | BIC | AIC | Ray-Turi | Davies-Bouldin |
| --- | --- | --- | --- | --- | --- |
| 2 | 4565.01 | -274916.8 | -274787.2 | -0.0719 | -1.45 |
| 3 | 5059.53 | -264020.6 | -263830.0 | -0.0786 | -1.53 |
| 4 | 5639.93 | -256472.7 | -256221.1 | -0.0977 | -1.49 |
| 5 | 5984.16 | -252224.9 | -251912.3 | -0.0860 | -1.45 |
| 6 | 5989.86 | -248341.5 | -247968.0 | -0.1128 | -1.49 |

*Note.* The Calinski-Harabasz index is the ratio of the sum of between-clusters variance and within-clusters variance. The higher the score, the denser and more separated clusters, thus the better the model fit.

Among these criteria, some should be maximized (high value denoting good partition) while others should be minimized (low value denoting good partition). To avoid this confusion, package *KmL3D* computes all criteria to be maximized. Thus, for all indices, a higher score indicates a better-fitting model.

# Table S8. Posterior probabilities of each trajectory.

| No. Clusters | Posterior Probability of Each Trajectory |
| --- | --- |
| 2 | 0.98, 0.79 |
| 3 | 0.97, 0.78, 0.91 |
| 4 | 0.96, 0.74, 0.93, 0.80 |
| 5 | 0.96, 0.77, 0.80, 0.94, 0.82 |
| 6 | 0.93, 0.79, 0.68, 0.83, 0.95, 0.85 |

Table S9. Distribution of sociodemographic characteristics and early risk factors across joint trajectories of bullying victimisation and perpetration from multiply imputed data.

|  |  |  |  | Mean (SD) or *n* (%) |  |  |
| --- | --- | --- | --- | --- | --- | --- |
|  | Age  measured | Uninvolved | Early Child Victims | Early Adolescent Victims | Early Child Bullies | Bully-Victims |
| Early Factor |  | (*n* = 8,706) | (*n* = 1,450) | (*n* = 2,195) | (*n* = 1,166) | (*n* = 1,047) |
| **Child** |  |  |  |  |  |  |
| Male sex*, n (%)* | 9 months | 4086 (47) | 782 (54) | 1114 (51) | 755 (65) | 680 (65) |
| Ethnicity, *n (%)* White | 9 months | 7403 (61) | 1128 (9) | 1932 (16) | 901 (7) | 872 (7) |
| Black or Black British |  | 250 (54) | 66 (14) | 41 (9) | 56 (12) | 47 (10) |
| Pakistani and Bangladeshi |  | 464 (52) | 130 (15) | 100 (11) | 121 (14) | 74 (8) |
| Indian |  | 231 (65) | 47 (13) | 37 (10) | 23 (6) | 17 (5) |
| Mixed |  | 223 (55) | 55 (14) | 55 (14) | 45 (11) | 24 (6) |
| Other (inc. Chinese) |  | 113 (63) | 20 (11) | 22 (12) | 14 (8) | 9 (5) |
| Birthweight, *g* | 9 months | 3.37 (0.58) | 3.31 (0.60) | 3.34 (0.61) | 3.29 (0.60) | 3.35 (0.60) |
| BMI, *kg/m²* | 3&5 yr. | 16.42 (1.95) | 16.60 (2.35) | 16.58 (1.94) | 16.52 (2.30) | 16.62 (1.99) |
| Physical health problem | 3&5 yr. | 0.25 (0.43) | 0.37 (0.48) | 0.28 (0.45) | 0.34 (0.47) | 0.29 (0.45) |
| Positive mood as an infant | 9 months | 19.26 (3.34) | 19.14 (3.58) | 19.25 (3.46) | 18.86 (3.57) | 19.22 (3.26) |
| Withdrawn as an infant | 9 months | 5.60 (2.49) | 5.80 (2.71) | 5.36 (2.38) | 5.86 (2.81) | 5.58 (2.52) |
| Unadaptable as an infant | 9 months | 8.59 (2.34) | 8.60 (2.54) | 8.41 (2.29) | 8.49 (2.59) | 8.32 (2.38) |
| Regular eating/sleeping as an infant | 9 months | 13.10 (2.17) | 12.72 (2.48) | 13.03 (2.28) | 12.53 (2.66) | 12.72 (2.55) |
| Independence and self-regulation | 3&5 yr. | 5.03 (0.57) | 4.91 (0.59) | 4.97 (0.57) | 4.80 (0.63) | 4.88 (0.60) |
| Emotional dysregulation | 3&5 yr. | 2.72 (0.78) | 3.05 (0.84) | 2.86 (0.83) | 3.45 (0.81) | 3.07 (0.86) |
| Cognitive ability | 5 yr. | 162.09 (22.52) | 155.03 (23.88) | 160.83 (23.75) | 151.06 (26.42) | 152.97 (26.38) |
| **Family** |  |  |  |  |  |  |
| Positive mother-child relationship | 3 yr. | 64.94 (6.50) | 62.86 (7.19) | 64.13 (6.95) | 59.79 (7.85) | 62.62 (7.33) |
| Harsh maternal discipline | 3&5 yr. | 33.22 (6.54) | 34.20 (6.80) | 34.34 (6.72) | 37.03 (6.80) | 35.28 (6.93) |
| Maternal mental health problems | 3&5 yr. | 11.76 (5.74) | 14.90 (7.43) | 12.53 (6.32) | 15.34 (7.75) | 13.55 (7.13) |
| Low family income | 9 months | 2.96 (1.39) | 3.49 (1.35) | 3.05 (1.40) | 3.77 (1.32) | 3.54 (1.38) |
| Family size, *n* | 3 yr. | 4.18 (1.21) | 4.14 (1.36) | 4.09 (1.22) | 4.30 (1.49) | 4.29 (1.47) |

# Table S10. Imputed data univariate regressions for associations between early child and family factors with bullying involvement trajectories.

|  | Early Child Victims | |  | Early Adolescent Victims | |  | Early Child Bullies | |  | Bully-Victims | |  |
| --- | --- | --- | --- | --- | --- | --- | --- | --- | --- | --- | --- | --- |
| Early Factor | *OR* | *95% CI* |  | *OR* | *95% CI* |  | *OR* | *95% CI* |  | *OR* | *95% CI* | |
| **Child** |  |  |  |  |  |  |  |  |  |  |  | |
| Male sex | 1.32 | 1.21-1.43*** |  | 1.17 | 1.08-1.27*** |  | 2.10 | 1.97-2.23*** |  | 2.11 | 1.97-2.24*** | |
| Birthweight | 0.84 | 0.74-0.93*** |  | 0.91 | 0.83-0.99* |  | 0.79 | 0.69-0.89*** |  | 0.94 | 0.83-1.05 | |
| BMI | 1.03 | 1.00-1.05* |  | 1.03 | 1.01-1.06** |  | 1.01 | 0.99-1.04 |  | 1.03 | 1.00-1.06* | |
| Physical health problem | 1.81 | 1.69-1.93*** |  | 1.22 | 1.12-1.33*** |  | 1.59 | 1.45-1.72*** |  | 1.23 | 1.08-1.37** | |
| Positive mood as an infant | 0.97 | 0.91-1.02 |  | 1.00 | 0.95-1.04 |  | 0.89 | 0.83-0.96*** |  | 0.99 | 0.92-1.05 | |
| Withdrawn as an infant | 1.08 | 1.03-1.14** |  | 0.90 | 0.85-0.95*** |  | 1.11 | 1.05-1.17*** |  | 0.99 | 0.93-1.06 | |
| Unadaptable as an infant | 1.00 | 0.95-1.06 |  | 0.93 | 0.88-0.98** |  | 0.96 | 0.90-1.02 |  | 0.89 | 0.82-0.96*** | |
| Regular eating/sleeping as an infant | 0.85 | 0.80-0.91*** |  | 0.97 | 0.92-1.02 |  | 0.79 | 0.73-0.85*** |  | 0.85 | 0.78-0.91*** | |
| Independence and self-regulation | 0.82 | 0.76-0.87*** |  | 0.89 | 0.84-0.94*** |  | 0.67 | 0.61-0.73*** |  | 0.77 | 0.71-0.84*** | |
| Emotional dysregulation | 1.49 | 1.44-1.55*** |  | 1.21 | 1.16-1.26*** |  | 2.49 | 2.42-2.55*** |  | 1.53 | 1.46-1.60*** | |
| Cognitive ability | 0.74 | 0.68-0.80*** |  | 0.94 | 0.89-0.99* |  | 0.63 | 0.57-0.69*** |  | 0.68 | 0.61-0.74*** | |
| **Family** |  |  |  |  |  |  |  |  |  |  |  | |
| Positive mother-child relationship | 0.74 | 0.68-0.79*** |  | 0.88 | 0.82-0.93*** |  | 0.51 | 0.45-0.57*** |  | 0.71 | 0.65-0.78*** | |
| Harsh maternal discipline | 1.14 | 1.09-1.20*** |  | 1.19 | 1.15-1.24*** |  | 1.82 | 1.75-1.88*** |  | 1.35 | 1.29-1.42*** | |
| Maternal mental health problems | 1.55 | 1.49-1.60*** |  | 1.15 | 1.10-1.20*** |  | 1.62 | 1.57-1.68*** |  | 1.31 | 1.24-1.37*** | |
| Low family income | 1.31 | 1.27-1.36*** |  | 1.04 | 1.01-1.08* |  | 1.55 | 1.50-1.60*** |  | 1.35 | 1.31-1.40*** | |
| Family size | 0.97 | 0.92-1.02 |  | 0.94 | 0.90-0.98** |  | 1.07 | 1.02-1.12** |  | 1.06 | 1.01-1.12* | |

*Note. Note. OR* = odds ratio; *CI* = confidence interval; BMI = body mass index.

**p* < .05; ***p* < .01; ****p* < .001 in univariate multinomial regression analyses with uninvolved children as the reference group.

# Table S11. Complete data univariate regressions for associations between early child and family factors with bullying involvement trajectories.

|  |  | Early Child Victims | |  | Early Adolescent Victims | |  | Early Child Bullies | |  | Bully-Victims | |
| --- | --- | --- | --- | --- | --- | --- | --- | --- | --- | --- | --- | --- |
| Early Factor | *n* | *OR* | *95% CI* |  | *OR* | *95% CI* |  | *OR* | *95% CI* |  | *OR* | *95% CI* |
| **Child** |  |  |  |  |  |  |  |  |  |  |  |  |
| Male sex | 14,564 | 1.32 | 1.18-1.48*** |  | 1.17 | 1.07-1.29*** |  | 2.10 | 1.85-2.39*** |  | 2.11 | 1.84-2.41*** |
| Birthweight | 14,522 | 0.84 | 0.76-0.92*** |  | 0.91 | 0.84-0.98* |  | 0.79 | 0.71-0.88*** |  | 0.94 | 0.84-1.05 |
| BMI | 14,564 | 1.03 | 1.01-1.05* |  | 1.03 | 1.01-1.06** |  | 1.01 | 0.99-1.04 |  | 1.03 | 1.00-1.06* |
| Physical health problem | 13,355 | 1.87 | 1.65-2.11*** |  | 1.23 | 1.10-1.36*** |  | 1.66 | 1.45-1.91*** |  | 1.26 | 1.08-1.46** |
| Positive mood as an infant | 14,143 | 0.96 | 0.91-1.02 |  | 1.00 | 0.95-1.05 |  | 0.89 | 0.83-0.94*** |  | 0.99 | 0.92-1.05 |
| Withdrawn as an infant | 14,113 | 1.08 | 1.02-1.15** |  | 0.90 | 0.86-0.95*** |  | 1.11 | 1.05-1.18*** |  | 0.99 | 0.93-1.06 |
| Unadaptable as an infant | 14,138 | 1.01 | 0.95-1.07 |  | 0.93 | 0.88-0.97** |  | 0.96 | 0.90-1.02 |  | 0.89 | 0.83-0.95*** |
| Regular eating/sleeping as an infant | 14,137 | 0.85 | 0.80-0.90*** |  | 0.97 | 0.92-1.01 |  | 0.79 | 0.75-0.84*** |  | 0.84 | 0.79-0.90*** |
| Independence and self-regulation | 14,386 | 0.81 | 0.77-0.86*** |  | 0.89 | 0.85-0.93*** |  | 0.67 | 0.63-0.71*** |  | 0.77 | 0.73-0.83*** |
| Emotional dysregulation | 14,386 | 1.51 | 1.42-1.59*** |  | 1.22 | 1.16-1.28*** |  | 2.55 | 2.39-2.73*** |  | 1.54 | 1.44-1.64*** |
| Cognitive ability | 14,345 | 0.74 | 0.70-0.78*** |  | 0.94 | 0.90-0.99* |  | 0.63 | 0.60-0.67*** |  | 0.68 | 0.64-0.72*** |
| **Family** |  |  |  |  |  |  |  |  |  |  |  |  |
| Positive mother-child relationship | 11,715 | 0.71 | 0.67-0.76*** |  | 0.86 | 0.81-0.91*** |  | 0.46 | 0.43-0.49*** |  | 0.69 | 0.64-0.74*** |
| Harsh maternal discipline | 14,012 | 1.15 | 1.08-1.22*** |  | 1.21 | 1.15-1.27*** |  | 1.96 | 1.82-2.10*** |  | 1.39 | 1.29-1.49*** |
| Maternal mental health problems | 13,995 | 1.59 | 1.50-1.68*** |  | 1.16 | 1.10-1.22*** |  | 1.68 | 1.58-1.78*** |  | 1.33 | 1.24-1.42*** |
| Low family income | 14,521 | 1.32 | 1.26-1.37*** |  | 1.04 | 1.01-1.08* |  | 1.55 | 1.48-1.63*** |  | 1.36 | 1.29-1.42*** |
| Family size | 13,160 | 0.96 | 0.91-1.00* |  | 0.94 | 0.90-0.98** |  | 1.07 | 1.02-1.13** |  | 1.07 | 1.01-1.12* |

# Table S12. Complete data multivariate regression for independent associations between early child and family factors with bullying involvement trajectories.

|  | Early Child Victims | |  | Early Adolescent Victims | |  | Early Child Bullies | |  | Bully-Victims | |
| --- | --- | --- | --- | --- | --- | --- | --- | --- | --- | --- | --- |
| Early Factor | *OR* | *95% CI* |  | *OR* | *95% CI* |  | *OR* | *95% CI* |  | *OR* | *95% CI* |
| **Child** |  |  |  |  |  |  |  |  |  |  |  |
| Male sex | 1.27 | 1.11-1.46*** |  | 1.12 | 1.01-1.25* |  | 1.90 | 1.60-2.25*** |  | 2.04 | 1.73-2.41*** |
| Birthweight | 0.89 | 0.79-1.00* |  | 0.88 | 0.80-0.96** |  | 0.91 | 0.79-1.04 |  | 0.94 | 0.82-1.07 |
| BMI | 1.04 | 1.01-1.08* |  | 1.04 | 1.01-1.07** |  | 1.05 | 1.01-1.09* |  | 1.03 | 0.99-1.07 |
| Physical health problem | 1.62 | 1.41-1.87*** |  | 1.20 | 1.07-1.35** |  | 1.20 | 1.01-1.42* |  | 1.04 | 0.87-1.23 |
| Positive mood as an infant | 1.05 | 0.98-1.13 |  | 1.01 | 0.95-1.06 |  | 0.98 | 0.91-1.06 |  | 1.05 | 0.97-1.14 |
| Withdrawn as an infant | 0.97 | 0.90-1.04 |  | 0.91 | 0.85-0.97** |  | 0.94 | 0.86-1.03 |  | 0.93 | 0.85-1.01 |
| Unadaptable as an infant | 1.07 | 1.00-1.16* |  | 0.95 | 0.89-1.01 |  | 1.00 | 0.91-1.09 |  | 0.94 | 0.86-1.03 |
| Regular eating/sleeping as an infant | 0.92 | 0.85-0.99* |  | 1.01 | 0.95-1.07 |  | 0.92 | 0.85-1.00* |  | 0.96 | 0.88-1.04 |
| Independence and self-regulation | 0.94 | 0.87-1.01 |  | 0.90 | 0.85-0.96*** |  | 0.93 | 0.85-1.02 |  | 0.94 | 0.86-1.02 |
| Emotional dysregulation | 1.27 | 1.17-1.38*** |  | 1.14 | 1.07-1.22*** |  | 1.86 | 1.68-2.05*** |  | 1.27 | 1.15-1.39*** |
| Cognitive ability | 0.89 | 0.83-0.96** |  | 0.99 | 0.93-1.05 |  | 0.91 | 0.83-0.99* |  | 0.85 | 0.78-0.93*** |
| **Family** |  |  |  |  |  |  |  |  |  |  |  |
| Positive mother-child relationship | 1.01 | 0.93-1.09 |  | 1.01 | 0.94-1.08 |  | 0.78 | 0.71-0.85*** |  | 0.94 | 0.86-1.03 |
| Harsh maternal discipline | 1.04 | 0.96-1.12 |  | 1.13 | 1.06-1.20*** |  | 1.48 | 1.35-1.62*** |  | 1.30 | 1.19-1.42*** |
| Maternal mental health problems | 1.49 | 1.38-1.60*** |  | 1.10 | 1.02-1.18** |  | 1.24 | 1.14-1.35*** |  | 1.12 | 1.02-1.23* |
| Low family income | 1.16 | 1.10-1.22*** |  | 1.04 | 1.00-1.08* |  | 1.31 | 1.23-1.40*** |  | 1.25 | 1.18-1.33*** |
| Family size | 0.93 | 0.88-0.99* |  | 0.98 | 0.94-1.03 |  | 1.06 | 1.00-1.13* |  | 1.04 | 0.98-1.11 |

*Note.* Sample size for complete cases was 11,338.

# Table S13. Multivariate regression with bully-victims coded as the reference group, conducted on multiply imputed data.

|  | Early Child Victims | |  | | Early Adolescent Victims | | |  | Early Child Bullies | |
| --- | --- | --- | --- | --- | --- | --- | --- | --- | --- | --- |
| Early Factor | *OR* | *95% CI* | |  | | *OR* | *95% CI* |  | *OR* | *95% CI* |
| **Child** |  |  | |  | |  |  |  |  |  |
| Male sex | 0.67 | 0.50-0.83*** | |  | | 0.58 | 0.42-0.74*** |  | 0.96 | 0.78-1.14 |
| Birthweight | 0.93 | 0.79-1.07 | |  | | 0.91 | 0.78-1.04 |  | 0.90 | 0.76-1.05 |
| BMI | 1.00 | 0.97-1.04 | |  | | 1.01 | 0.97-1.04 |  | 0.99 | 0.96-1.03 |
| Physical health problem | 1.51 | 1.33-1.69*** | |  | | 1.09 | 0.92-1.26 |  | 1.16 | 0.97-1.35 |
| Positive mood as an infant | 0.99 | 0.90-1.07 | |  | | 0.97 | 0.90-1.05 |  | 0.95 | 0.86-1.04 |
| Withdrawn as an infant | 1.05 | 0.96-1.14 | |  | | 0.96 | 0.88-1.05 |  | 1.05 | 0.95-1.14 |
| Unadaptable as an infant | 1.11 | 1.02-1.20* | |  | | 1.02 | 0.93-1.10 |  | 1.04 | 0.95-1.14 |
| Regular eating/sleeping as an infant | 0.98 | 0.89-1.06 | |  | | 1.05 | 0.97-1.13 |  | 0.98 | 0.90-1.07 |
| Independence and self-regulation | 1.03 | 0.95-1.12 | |  | | 1.01 | 0.93-1.09 |  | 0.99 | 0.90-1.08 |
| Emotional dysregulation | 1.01 | 0.92-1.11 | |  | | 0.93 | 0.84-1.02 |  | 1.42 | 1.32-1.52*** |
| Cognitive ability | 1.08 | 1.00-1.17* | |  | | 1.22 | 1.13-1.30*** |  | 1.05 | 0.96-1.14 |
| **Family** |  |  | |  | |  |  |  |  |  |
| Positive mother-child relationship | 1.06 | 0.96-1.16 | |  | | 1.08 | 0.99-1.18 |  | 0.89 | 0.78-0.99* |
| Harsh maternal discipline | 0.83 | 0.75-0.92*** | |  | | 0.91 | 0.83-0.99* |  | 1.17 | 1.08-1.26*** |
| Maternal mental health problems | 1.23 | 1.15-1.31*** | |  | | 0.99 | 0.91-1.07 |  | 1.08 | 0.99-1.16 |
| Low family income | 0.95 | 0.88-1.01 | |  | | 0.82 | 0.76-0.88*** |  | 1.06 | 0.99-1.14 |
| Family size | 0.92 | 0.85-0.98** | |  | | 0.93 | 0.87-0.99* |  | 1.02 | 0.95-1.08 |

# Table S14. Multivariate regression with bully-victims coded as the reference group, conducted on complete cases.

|  | Early Child Victims | |  | | Early Adolescent Victims | | |  | Early Child Bullies | |
| --- | --- | --- | --- | --- | --- | --- | --- | --- | --- | --- |
| Early Factor | *OR* | *95% CI* | |  | | *OR* | *95% CI* |  | *OR* | *95% CI* |
| **Child** |  |  | |  | |  |  |  |  |  |
| Male sex | 0.62 | 0.51-0.76*** | |  | | 0.55 | 0.46-0.66*** |  | 0.93 | 0.75-1.16 |
| Birthweight | 0.95 | 0.81-1.12 | |  | | 0.94 | 0.81-1.09 |  | 0.97 | 0.82-1.16 |
| BMI | 1.01 | 0.96-1.06 | |  | | 1.01 | 0.96-1.05 |  | 1.02 | 0.97-1.07 |
| Physical health problem | 1.56 | 1.27-1.92*** | |  | | 1.16 | 0.95-1.40 |  | 1.15 | 0.92-1.44 |
| Positive mood as an infant | 1.00 | 0.91-1.11 | |  | | 0.96 | 0.88-1.05 |  | 0.94 | 0.84-1.04 |
| Withdrawn as an infant | 1.04 | 0.94-1.16 | |  | | 0.98 | 0.89-1.09 |  | 1.02 | 0.90-1.14 |
| Unadaptable as an infant | 1.14 | 1.02-1.27* | |  | | 1.00 | 0.91-1.11 |  | 1.06 | 0.94-1.18 |
| Regular eating/sleeping as an infant | 0.96 | 0.87-1.06 | |  | | 1.05 | 0.96-1.16 |  | 0.96 | 0.87-1.07 |
| Independence and self-regulation | 1.00 | 0.90-1.11 | |  | | 0.96 | 0.87-1.06 |  | 1.00 | 0.89-1.12 |
| Emotional dysregulation | 1.00 | 0.89-1.12 | |  | | 0.90 | 0.81-1.00* |  | 1.47 | 1.29-1.66*** |
| Cognitive ability | 1.05 | 0.94-1.16 | |  | | 1.17 | 1.06-1.28** |  | 1.06 | 0.95-1.19 |
| **Family** |  |  | |  | |  |  |  |  |  |
| Positive mother-child relationship | 1.07 | 0.96-1.20 | |  | | 1.08 | 0.97-1.20 |  | 0.83 | 0.74-0.93** |
| Harsh maternal discipline | 0.80 | 0.72- 0.89*** | |  | | 0.87 | 0.79-0.96** |  | 1.14 | 1.02-1.28* |
| Maternal mental health problems | 1.32 | 1.19-1.47*** | |  | | 0.98 | 0.88-1.09 |  | 1.10 | 0.99-1.23 |
| Low family income | 0.93 | 0.86-1.00* | |  | | 0.83 | 0.78-0.89*** |  | 1.05 | 0.96-1.14 |
| Family size | 0.89 | 0.83-0.97** | |  | | 0.94 | 0.87-1.01 |  | 1.02 | 0.94-1.10 |

# Figure S1. The five joint trajectories of bullying victimisation and perpetration.

# Figure S2. Three-dimensional graphs of mean joint trajectories with time on the x-axis, perpetration on the y-axis, and victimisation on the z-axis.

#

# Figure S3. Six-trajectory solution of the joint trajectories of victimisation and perpetration.

Sensitivity Analyses

As sensitivity analyses, we estimated the joint trajectories of victimisation and perpetration using single informants: parent reports (mothers rated whether their child was bullied or bullied others at ages 5, 7, and 14 years), and child reports (children self-reported whether they were bullied or bullied others at ages 7, 11, and 14). We were not able to derive trajectories from teacher reports as teacher reports were only available at two time points (ages 7 and 11).

For the parent-reported trajectory model, average posterior probabilities were the highest for the five- and six-trajectory solutions (Table S15). The model-fitting indices suggested that the optimal number of trajectories was six (supported by Calinski-Harabasz, AIC, and BIC), with the five-trajectory solution identified as the second-best choice on the Calinski-Harabasz, Ray-Turi, AIC, and BIC indices (Table S16). However, the five-trajectory solution was most consistent with prior studies modelling joint trajectories of victimisation and perpetration as it also derived uninvolved children, bullies, a decreasing victimisation trajectory (Haltigan & Vaillancourt), an increasing victimisation trajectory (Barker et al., 2008), as well as bully-victims with high/increasing victimisation and high/increasing perpetration (Barker et al., 2008; Walters 2021; Zhou et al., 2020). Given that the five-trajectory solution was most consistent with past evidence, had the joint highest posterior probabilities and also had high scores on model-fitting indices, we selected this model for parent-reported trajectories.

# Table S15. Posterior probabilities for parent-reported trajectory solutions.

| No. Clusters | Posterior Probability of Each Trajectory |
| --- | --- |
| 2 | 0.99, 0.76 |
| 3 | 0.98, 0.76, 0.88 |
| 4 | 0.98, 0.79, 0.92, 0.97 |
| 5 | 0.96, 0.89, 0.91, 0.97, 0.98 |
| 6 | 0.94, 0.93, 0.92, 0.91, 0.99, 0.97 |

# Table S16. Model-fitting indices for parent-reported trajectory solutions.

| No. Clusters | Calinski-Harabasz | BIC | AIC | Ray-Turi | Davies-Bouldin |
| --- | --- | --- | --- | --- | --- |
| 2 | 6309.63 | -200782.3 | -200683.0 | -0.05 | -1.27 |
| 3 | 8651.32 | -194006.7 | -193861.5 | -0.09 | -1.29 |
| 4 | 8685.86 | -185947.7 | -185756.8 | -0.08 | -1.36 |
| 5 | 9549.70 | -177816.9 | -177580.1 | -0.07 | -1.32 |
| 6 | 9907.97 | -175199.5 | -174916.9 | -0.08 | -1.30 |

*Note.* As *KmL3D* computes all criteria to be maximized, for all indices a higher score indicates a better-fitting model.

For the child-reported trajectory model, the average posterior probability was highest for the five-trajectory solution (Table S17). The model-fitting criteria suggested the optimal number of trajectories was six (supported by Calinski-Harabasz, AIC, BIC, Davies-Bouldin) with the five-trajectory solution identified as the second-best choice on the Calinski-Harabasz, Davies-Bouldin, AIC, and BIC indices (Table S18). However, the five-trajectory solution was most consistent with prior studies modelling joint trajectories of victimisation and perpetration as it also derived uninvolved children, a decreasing victimisation trajectory (Haltigan & Vaillancourt, 2014), a chronic victim trajectory from childhood to adolescence (Zhou et al., 2020), as well as one increasing bully-victim group and one decreasing bully-victim group (Walters, 2021; Zhou et al., 2020). Given that the five-trajectory solution was most consistent with previous evidence and had the highest posterior probabilities and high model-fitting indices, we selected this model for child-reported trajectories.

# Table S17. Posterior probabilities for child-reported trajectory solutions.

| No. Clusters | Posterior Probability of Each Trajectory |
| --- | --- |
| 2 | 0.97, 0.80 |
| 3 | 0.97, 0.73, 0.86 |
| 4 | 0.96, 0.73, 0.91, 0.83 |
| 5 | 0.90, 0.84, 0.93, 0.93, 0.93 |
| 6 | 0.91, 0.86, 0.96, 0.88, 0.96, 0.86 |

# Table S18. Model-fitting indices for child-reported trajectory solutions.

| No. Clusters | Calinski-Harabasz | BIC | AIC | Ray-Turi | Davies-Bouldin |
| --- | --- | --- | --- | --- | --- |
| 2 | 5004.45 | -190694.7 | -190596.7 | -0.09 | -1.37 |
| 3 | 6336.97 | -185837.6 | -185694.3 | -0.12 | -1.37 |
| 4 | 6740.48 | -178020.2 | -177831.7 | -0.11 | -1.37 |
| 5 | 7537.94 | -173508.8 | -173275.1 | -0.18 | -1.30 |
| 6 | 7913.48 | -163388.0 | -163109.0 | -0.15 | -1.29 |

*Comparability of parent and child-reported trajectories with multi-informant trajectories*

Like the multi-informant trajectories, parent-reported trajectories (age 5-14 years) included groups of uninvolved children, adolescent victims, child victims, child bullies, and bully-victims (Figure S4). Child-reported trajectories (age 7-14 years) included groups of uninvolved children, child victims, chronic victims (peaking in early adolescence), child bully-victims, and adolescent bully-victims (Figure S5). Therefore, the child-reported trajectories were similar to, but not exactly the same as the multi-informant and parent-reported trajectories. This is likely to be in part because the measurement period (age 7-14) was not entirely consistent with period covered by the multi-informant trajectories (age 5-14 years). However, across all informant-reported trajectories, we observed broadly consistent patterns of uninvolved children, child victims, victims with highest levels in early adolescence, and bully-victims.

# Figure S4. Parent-reported five-trajectory solution.


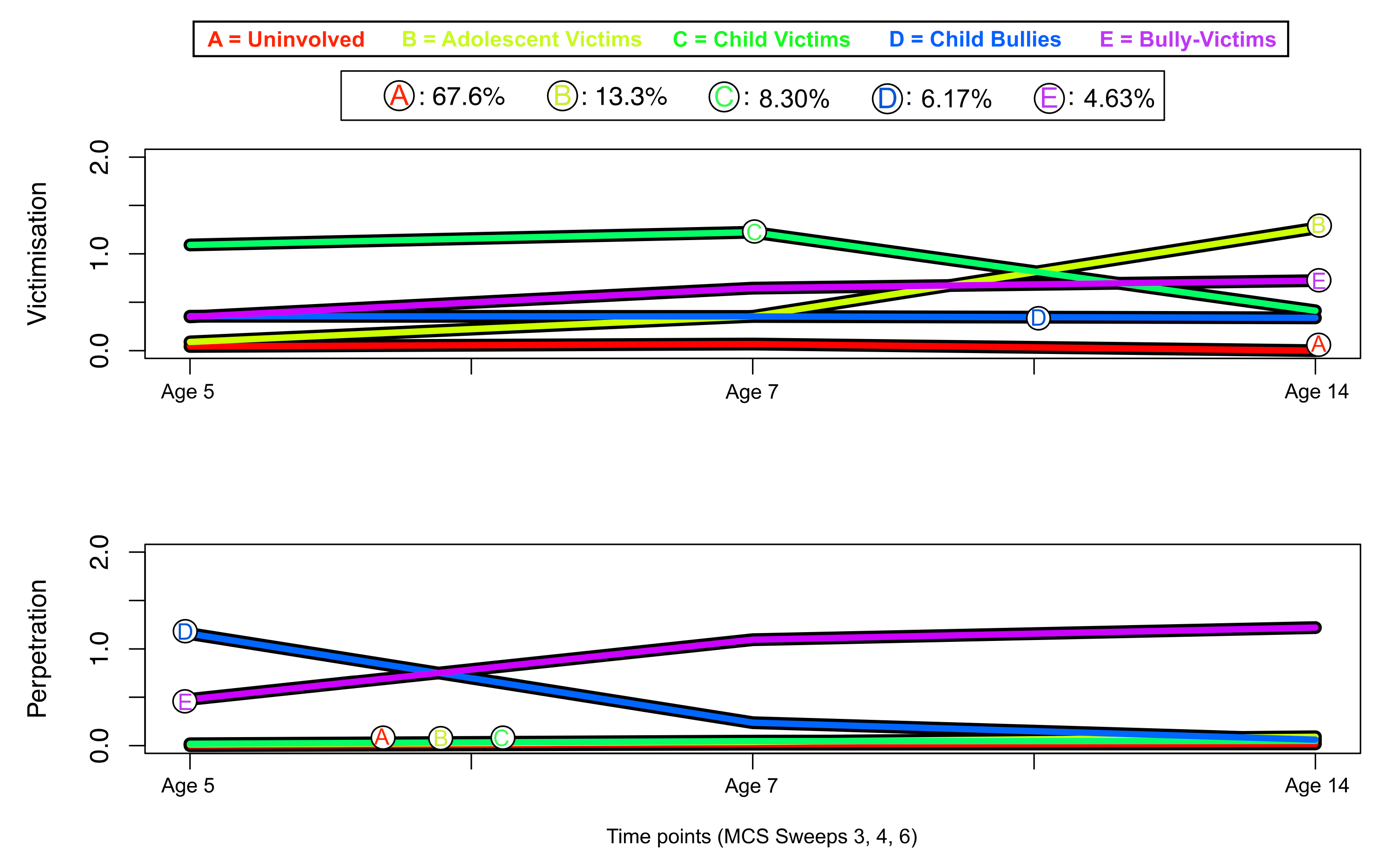


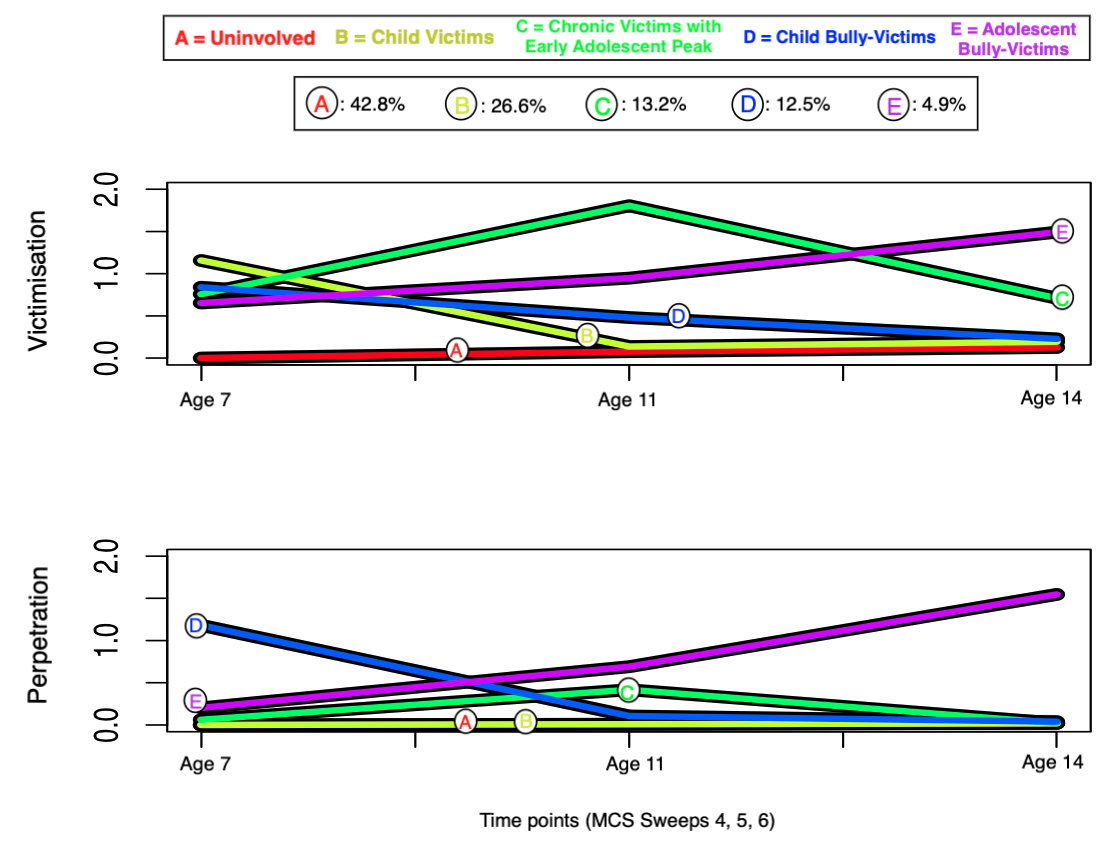
Figure S5. Child-reported five-trajectory solution.

*Sensitivity analysis according to missing data requirements*

As a further sensitivity analysis, we reran the trajectories using a different missing data requirement. Our main analyses modelled missing data using the *CopyMean* method with maxNA=3, where participants were required to have data for at least one out of four time points. To check the effect of using more than one data point, we set maxNA=2, where participants were required to have data for at least two out of four time points (N=13,934). The five-trajectory solution (Figure S6) produced very similar results to that in the main analyses, as groups included uninvolved children (n=8,108 [58.2%]), early adolescent victims (n=2,281 [16.4%]), early child victims (n=1,361 [9.77%]), early child bullies (n=1,121 [8.05%]), and bully-victims (n=1,063 [7.63%]). For comparison, in our original analysis (where participants needed 1 data point to be included), the groups included uninvolved children (n=8,706 [59.78%]), early adolescent victims (n=2,195 [15.07%]), early child victims (n=1,450 [9.96%]), early child bullies (n=1,166 [8.01%]), and bully-victims (n=1,047 [7.19%]).

#
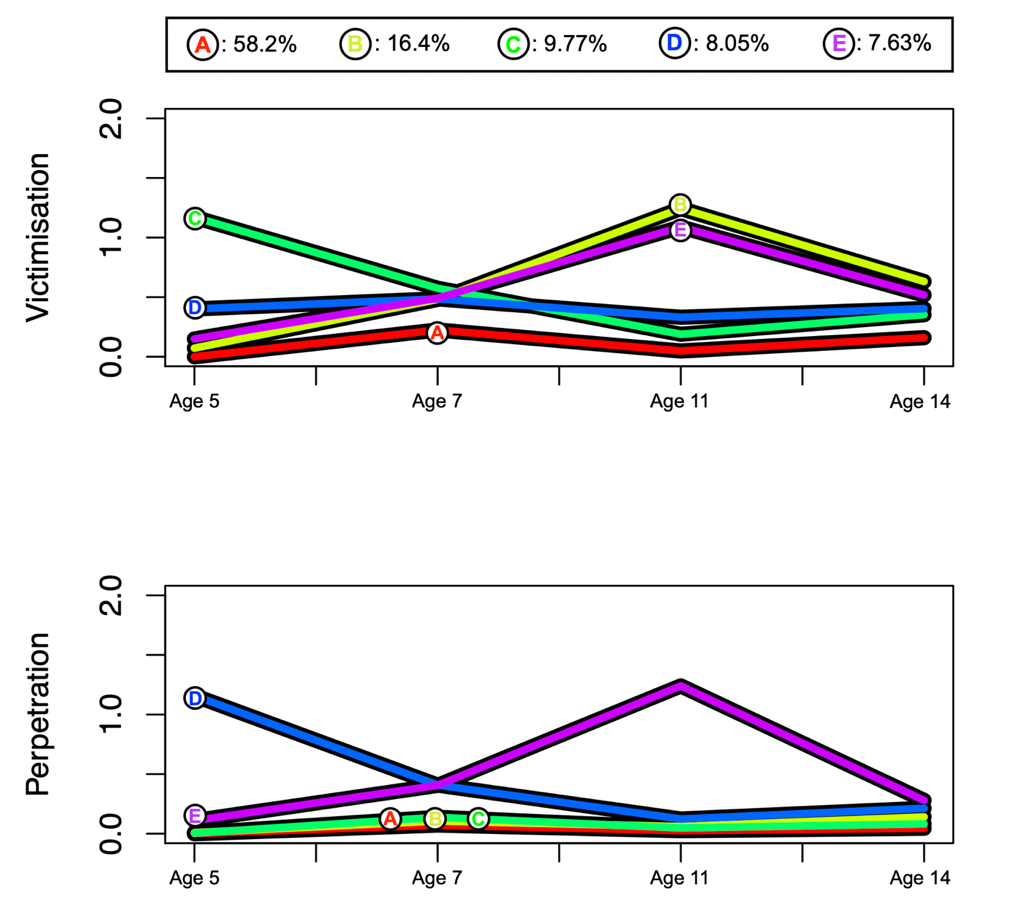
Figure S6. Multi-informant five-trajectory solution, when we used a different missing data requirement (maxNA=2).


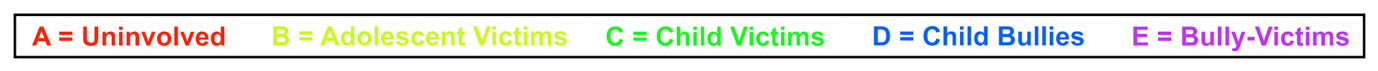


References

Akaike, H. (1974). A new look at the statistical model identification. *IEEE Transactions on Automatic Control*, *19*(6), 716–723. https://doi.org/10.1109/TAC.1974.1100705

Barker, E. D., Arseneault, L., Brendgen, M., Fontaine, N., & Maughan, B. (2008). Joint development of bullying and victimisation in adolescence: Relations to delinquency and self-Harm. *Journal of the American Academy of Child & Adolescent Psychiatry*, *47*(9), 1030–1038. https://doi.org/10.1097/CHI.ObO13e31817eec98

Bolstad, W. (2007). *Introduction to Bayesian Statistics* (2nd ed.). John Wiley & Sons, Inc.

Caliński, T., & Harabasz, J. (1974). A dendrite method for cluster analysis. *Communciations in Statistics*, *3*(1), 1–27. https://doi.org/10.1080/03610927408827101

Carey, W. B., & McDevitt, S. C. (1978). Revision of the Infant Temperament Questionnaire. *Pediatrics*, *61*(5), 735–739.

Davies, D. L., & Bouldin, D. W. (1979). A cluster separation measure. *IEEE Transactions on Pattern Analysis and Machine Intelligence*, *1*(2), 224–227. https://doi.org/10.1109/TPAMI.1979.4766909

Elliott, C. D., Smith, P., & McCulloch, K. (1996). *British Ability Scales Second Edition (BAS II). Administration and Scoring Manual.* Nelson.

Haltigan, J. D., & Vaillancourt, T. (2014). Joint trajectories of bullying and peer victimisation across elementary and middle school and associations with symptoms of psychopathology. *Developmental Psychology*, *50*(11), 2426–2436. https://doi.org/10.1037/a0038030

Hogan, A. E., Scott, K. G., & Bauer, C. R. (1992). The adaptive social behavior inventory (ASBI): A new assessment of social competence in high-risk three-year-olds. *Journal of Psychoeducational Assessment*, *10*, 230–239. https://doi.org/10.1177/073428299201000303

Kessler, R. C., Barker, P. R., Colpe, L. J., Epstein, J. F., Gfroerer, J. C., Hiripi, E., Howes, M. J., Normand, S.-L. T., Manderscheid, R. W., Walters, E. E., & Zaslavsky, A. M. (2003). Screening for serious mental illness in the general population. *Archives of General Psychiatry*, *60*(2), 184–189. https://doi.org/doi:10.1001/archpsyc.60.2.184

Pianta, R. C. (1992). *Child-Parent Relationship Scale*. University of Virginia.

Ray, S., & Turi, R. H. (1999). Determination of Number of Clusters in K -Means Clustering and Application in Colour Image Segmentation. *Proceedings of the 4th International Conference on Advances in Pattern Recognition and Digital Techniques*, 137–143.

Schwarz, G. (1978). Estimating the dimension of a model. *The Annals of Statistics*, *6*(2), 461–464. https://doi.org/10.1214/aos/1176344136

Straus, M. A., & Hamby, S. L. (1997). Measuring physical and psychological maltreatment of children with the conflict tactics scales. In G. K. Kantor & J. L. Jasinski (Eds.), *Out of the darkness: Contemporary research perspectives on family violence* (pp. 119–135). Sage Publications, Inc.

van Lier, P. A. C., van der Ende, J., Koot, H. M., & Verhulst, F. C. (2007). Which better predicts conduct problems? The relationship of trajectories of conduct problems with ODD and ADHD symptoms from childhood into adolescence. *Journal of Child Psychology and Psychiatry*, *48*(6), 601–608. https://doi.org/10.1111/j.1469-7610.2006.01724.x

Walters, G. D. (2021). Trajectories of bullying victimisation and perpetration in Australian school children and their relationship to future delinquency and conduct problems. *Psychology of Violence, 11*(1), 19–27. https://doi.org/10.1037/vio0000322

Zhou, Y., Zheng, H., Liang, Y., Wang, J., Han, R., & Liu, Z. (2020). Joint developmental trajectories of bullying and victimisation from childhood to adolescence: A parallel-process latent class growth analysis. *Journal of Interpersonal Violence*, *6*, 1–25. https://doi.org/10.1177/0886260520933054
